# Comparing longitudinal CD4 responses to cART among non-perinatally HIV-infected youth versus adults: Results from the HIVRN Cohort

**DOI:** 10.1371/journal.pone.0171125

**Published:** 2017-02-09

**Authors:** Allison L. Agwu, John A. Fleishman, Guy Mahiane, Bareng Aletta Sanny Nonyane, Keri N. Althoff, Baligh R. Yehia, Stephen A. Berry, Richard Rutstein, Ank Nijhawan, Christopher Mathews, Judith A. Aberg, Jeanne C. Keruly, Richard D. Moore, Kelly A. Gebo, for the HIV Research Network

**Affiliations:** 1 Division of Pediatric Infectious Diseases, Department of Pediatrics, Johns Hopkins School of Medicine, Baltimore, MD, United States of America; 2 Division of Infectious Diseases, Department of Medicine, Johns Hopkins School of Medicine, Baltimore, MD, United States of America; 3 Center for Financing, Access, and Cost Trends, Agency for Healthcare Research and Quality, Rockville, MD, United States of America; 4 Department of Biostatistics, Johns Hopkins Bloomberg School of Public Health, Baltimore, MD, United States of America; 5 Department of International Health, Johns Hopkins Bloomberg School of Public Health, Baltimore, MD, United States of America; 6 Bloomberg School of Public Health, Johns Hopkins Medical Institutions, Baltimore, MD, United States of America; 7 Division of Infectious Diseases, Department of Medicine, University of Pennsylvania School of Medicine, Philadelphia, PA, United States of America; 8 Division of General Pediatrics, Children’s Hospital of Philadelphia, Philadelphia, PA, United States of America; 9 Department of Internal Medicine, UT Southwestern Medical Center, Parkland Health and Hospital System, Dallas TX, United States of America; 10 Department of Medicine, University of California San Diego, San Diego, CA, United States of America; 11 Department of Medicine, Division of Infectious Diseases, Icahn School of Medicine at Mount Sinai, New York, NY, United States of America; 12 Division of General Internal Medicine, Department of Medicine, Johns Hopkins School of Medicine, Baltimore, MD, United States of America; Rush University, UNITED STATES

## Abstract

**Background:**

Youth have residual thymic tissue and potentially greater capacity for immune reconstitution than adults after initiation of combination antiretroviral therapy (cART). However, youth face behavioral and psychosocial challenges that may make them more likely than adults to delay ART initiation and less likely to attain similar CD4 outcomes after initiating cART. This study compared CD4 outcomes over time following cART initiation between ART-naïve non-perinatally HIV-infected (nPHIV) youth (13–24 years-old) and adults (≥25–44 years-old).

**Methods:**

Retrospective analysis of ART-naïve nPHIV individuals 13–44 years-old, who initiated their first cART between 2008 and 2011 at clinical sites in the HIV Research Network. A linear mixed model was used to assess the association between CD4 levels after cART initiation and age (13–24, 25–34, 35–44 years), accounting for random variation within participants and between sites, and adjusting for key variables including gender, race/ethnicity, viral load, gaps in care (defined as > 365 days between CD4 tests), and CD4 levels prior to cART initiation (baseline CD4).

**Results:**

Among 2,595 individuals (435 youth; 2,160 adults), the median follow-up after cART initiation was 179 weeks (IQR 92–249). Baseline CD4 was higher for youth (320 cells/mm^3^) than for ages 25–34 (293) or 35–44 (258). At 239 weeks after cART initiation, median unadjusted CD4 was higher for youth than adults (576 vs. 539 and 476 cells/mm^3^, respectively), but this difference was not significant when baseline CD4 was controlled. Compared to those with baseline CD4 ≤200 cells/mm^3^, individuals with baseline CD4 of 201–500 and >500 cells/mm^3^ had greater predicted CD4 levels: 390, 607, and 831, respectively. Additionally, having no gaps in care and higher viral load were associated with better CD4 outcomes.

**Conclusions:**

Despite having residual thymic tissue, youth attain similar, not superior, CD4 gains as adults. Early ART initiation with minimal delay is as essential to optimizing outcomes for youth as it is for their adult counterparts.

## Introduction

Thirty years into the HIV epidemic, effective combination antiretroviral therapy (cART) has transformed HIV into a chronic disease. Life expectancy for persons living with HIV (PLHIV) has improved dramatically, mirroring that of uninfected adults. [1,2] However, non-perinatally HIV-infected (nPHIV) youth between the ages of 13 and 24 comprise a unique and challenging group, in which the incidence of HIV infection is increasing. [3] Youth tend to have higher CD4 counts than adults when presenting for care, though 40% have CD4 counts below 350 cells/mm^3^ at presentation. [4,5] [6–10]

Increasingly, studies support earlier initiation of cART [11,12] and guidelines now recommend initiation of cART for all PLHIV, regardless of CD4 count. [13,14] However, studies have shown that youth are less likely than adults to initiate cART when meeting treatment criteria. [5,15] Age, their cognitive developmental stage (concrete thinking and invincibility), and their high rates of social marginalization and stigma, make diagnosing, engaging, and treating youth difficult. [16–18] For example, youth have higher rates of nonadherence to cART and attrition from care than adults. [6–10] Youth have residual thymic tissue and may potentially have greater capacity for immune reconstitution than adults. [19–23] However, it is not clear if the potential for enhanced CD4 recovery for youth, given their residual thymic tissue, compensates for their delays in initiating cART.

Unfortunately, studies examining CD4 responses in PLHIV have had limited capacity to look specifically at youth, as they commonly use broad age ranges (e.g., 18–39) and do not distinguish youth. [21,24,25] Further, other studies of youth have been conducted in clinical trials; generalization to non-trial settings may be limited. [8,26,27] In this study, we compared CD4 responses following cART initiation between nPHIV youth (13–24 years-old) and adults (≥25–44 years-old) followed in a large observational clinical cohort.

## Methods

This retrospective study compared trends in CD4 levels over time after initiation of cART between nPHIV youth aged 13–24 and adults aged 25 through 44 years at study entry. Studies that have examined differences in immunologic responses between young and older adults have used varying age comparisons,[28–30] e.g., 18–30 vs. 30–40, 40–50, 50-<60 and ≥60 [31], ≥16-<32.7 vs. 32.7–37.4, 27.2–44.4, and ≥44.5 [32], <50 vs. ≥50 years of age. [24,33] Differences between younger and older adults tended to emerge when comparing younger individuals with those ≥50 [24], with a few studies finding differences when comparing younger individuals to those > = 44.5 and 40–50 years of age.[31,32] Notably, most of the studies lumped all younger adults (e.g., 18–49) and had limited numbers of the youngest patients (i.e., <24), making it challenging to decipher differences in immune responses to cART among the youngest age groups. We therefore intentionally chose 45 as the upper age cutoff for our study to specifically compare CD4 response to cART among individuals where it would least be expected, by the cumulative evidence, that there should be differences and where any differences seen would not be attributed to the expected changes with older age.

Eligible patients were recruited from those treated in HIV clinics participating in the HIV Research Network (HIVRN), a consortium of 19 U.S. clinic sites that provide primary and subspecialty care to HIV-infected patients.[34,35] As described previously, HIVRN sites abstract specified data elements from patients’ medical records, including demographic data, service utilization, medications, and laboratory tests; abstracted data are assembled into a single database after quality assurance review. [34,35] The study was approved by the Johns Hopkins University School of Medicine Institutional review board (IRB) and the IRBs of each participating institution.

### Eligibility criteria

Patients were eligible if they were at least 12 years old but less than 45 years old, enrolled in the HIVRN clinic between January 1, 2008 and December 31, 2011, acquired HIV through risk behaviors (e.g., sexual activity, injection drug use), were ART-naïve at clinic enrollment, actively receiving HIV care (defined as having at least one CD4 count and one outpatient HIV provider visit within a calendar year), and initiated cART at some point between enrollment and December 31, 2011. In the analytic sample, the minimum age was 13. To focus on a therapy-naïve group, participants who had prior history of ART usage and whose first recorded HIV-1 RNA test was ≤400 copies/mL were excluded.

### Data collection and measures

Demographic and clinical data were collected from the HIVRN clinical database. CART was defined as concomitant use of ≥ 3 antiretroviral drugs either from ≥ 2 classes (nucleoside/nucleotide reverse transcriptase inhibitors (NRTIs), non-nucleoside reverse transcriptase inhibitors, protease inhibitors (PIs), integrase inhibitors, and entry inhibitors) or 3 NRTIs that qualified as cART (i.e., abacavir, lamivudine, and zidovudine). The date of initiation of cART was also recorded. Initial cART therapy was categorized as NNRTI, PI, other, or unknown.

Each HIVRN site recorded the dates and outcomes of all CD4 tests performed during the observation period of Jan 1, 2008 –December 31, 2014. The baseline CD4 test was the one performed closest in time prior to the cART initiation date and ≤16 weeks prior to cART initiation. If a patient had no recorded CD4 prior to cART initiation, a CD4 test performed up to 7 days after cART initiation was used. Patients with no CD4 test in the appropriate time window to establish a baseline were excluded. Baseline CD4 level was categorized as ≤200, 201–500, and >500 cells/mm^3^.

The age when each patient initiated cART was calculated from month and year of birth and the date of cART initiation. Age was categorized as 13–24, 25–34, and 35–44 years. Self-identified racial/ethnic group was classified as non-Hispanic White, non-Hispanic Black, Hispanic, or “other” (American Indian or Alaskan Native, Asian or Pacific Islander). HIV transmission group was classified as men who have sex with men (MSM), injection drug use (IDU), heterosexual activity (HET), or other.

Dates and results of all HIV-1 RNA (viral load (VL)) tests during the observation period were recorded. For analysis, VL values were categorized as ≤400, 401–1000, and >1000 copies/mL. VL tests were matched with CD4 tests by date. Most (87%) CD4 tests had a VL test on the same date. If no exact match by date existed, the VL test closest in time to the CD4 test (preferentially prior) was used. For the 3,270 CD4 records without an exact match to VL date, the median number of days between CD4 and VL tests was 1 day prior (IQR: 8 days prior to 32 days after).

As an indicator of gaps in care following cART initiation, we derived a dichotomous variable, “year_gap,” that equaled 1 if more than 365 days had elapsed between a CD4 test and the CD4 test immediately prior. Follow-up data on relevant clinical and demographic variables were available through December 31, 2014. All patient data were included until CD4 test data ended, due to death, elective withdrawal from HIVRN cohort, or the end of the observation period.

### Statistical analysis

The primary aim of the analysis was to examine the extent of increase in CD4 levels over time, subsequent to initiation of cART, and to assess whether age groups differed in their rates of increase, especially comparing those aged 13–24 to older adults. We distinguish the “baseline” CD4, which is the CD4 test immediately prior to cART initiation, from the outcome variables, which are CD4 tests subsequent to cART initiation. Analyses included all CD4 tests post-cART initiation and within the observation period, regardless of changes in ART regimen or nonadherence. To provide a context for interpreting the CD4 results, and given the critical importance of adherence as measured by virologic suppression, we also examined change over time in viral load.

Longitudinal analyses of CD4 levels used a linear mixed model. Because each patient contributed multiple CD4 measures over time, CD4 outcome measures for a single patient were not independent. Further, because patients were clustered in clinics, observations on patients in the same clinic were not independent. The three-level mixed model (CD4 tests, patients, and clinics) included random effects for clinic and for patient, thereby adjusting for correlated observations at these levels. In addition to age group, the model adjusted for initial treatment regimen, gender, race/ethnicity, HIV acquisition risk, year when cART was initiated, and baseline CD4 level. Viral load and “year_gap” were included as time-varying covariates. Of note, as the focus was CD4 outcome, VL temporally close to each CD4 measurement was used in the analysis rather than baseline VL, as this was thought, a priori, to be the more relevant VL measure associated with CD4, as it is a surrogate for adherence.

To assess CD4 trends, the time origin was the cART initiation date, and the time metric was time since cART initiation; this allows for comparisons of participants at the same time since cART initiation. The statistical model thus included a variable reflecting the number of weeks between the date of cART initiation and the dates of subsequent CD4 tests. To facilitate comparison with prior studies, which examined CD4 outcomes as 24 and 48 weeks [8,26,36], the time variable was scaled in 24-week units; thus, a time value of “2” represents 48 weeks from baseline. To incorporate potential non-linearity in the CD4 time trend, the model included both linear and squared time variables. To incorporate patient-level variation in CD4 time trends, both time variables had random effects, which were correlated. To incorporate age differences in time trends, the model included interactions between age group and each time variable.

We estimated two models. The first excluded baseline CD4, and the second included it. Comparing the models provides a sense of the degree to which effects of time-constant characteristics (e.g., gender, race/ethnicity) are mediated by baseline CD4. The second model is the main model in the analysis. To reflect differential time trends as a function of baseline CD4, the second model also included an interaction between the linear time variable and baseline CD4 category. The second model also included an interaction between age group and baseline CD4 category in order to evaluate whether the CD4 response of youth vs. adults is related to the baseline CD4 level. The baseline CD4 was not itself included in the set of CD4 outcomes, which began with the first CD4 test after cART initiation. A third analysis included a three-way interaction between time, age group, and baseline CD4; this interaction was not significant and these results are not presented. Patients with no CD4 tests recorded after cART initiation, or whose first CD4 test occurred after cART initiation, were excluded from analyses.

To help interpret the results of the second model, we calculated mean predicted outcomes for interactions between (1) time since cART initiation and age group and (2) age group and baseline CD4, and (3) time since cART initiation and baseline CD4.

Finally, to examine change in VL by age group, we conducted a sensitivity analysis that examined the proportion in each group who were suppressed (≤ 400 copies/mL) in each 24-week period. We also estimated a multilevel logistic model including time, age group, and their interaction. Analyses were performed using Stata Version 13 (StataCorp, TX), using a p-value of <0.05.

## Results

Initially, 3,110 individuals were eligible for analysis. Of these, we excluded 246 (8%) whose baseline CD4 test occurred more than 16 weeks before the start of cART, 206 (6%) who had no CD4 test after cART, 58 (2%) whose first recorded CD4 test was after cART initiation, and 5 (<1%) missing all VL data.

For the remaining 2,595 patients, the median number of weeks in the observation period (between the cART initiation date and the last CD4 test) was 179 (IQR = 92–249 weeks). The median number of CD4 measures post-cART initiation was 9 (IQR = 4–14). Fifty-eight patients (2%) died during the observation period. The 2,595 patients contributed a total of 25,334 CD4 tests, of which 41 were excluded due to missing timely viral load data and another 17 due to missing CD4 test results, resulting in 25,276 CD4 measures in the analysis.

Of 2,595 patients, 435 (17%) were youth aged 13–24, but only 12 patients were aged < 18. Compared to adults, youth had significantly higher proportions of patients who were male, black, MSM, with baseline CD4 above 200 cells/mm^3^, and receiving an initial NNRTI regimen. (Table 1) The mean number of weeks in the observation period was similar across age groups: 171, 169, and 177, from the youngest to the oldest, respectively. *CD4 analysis*.

**Table 1:** Sample Description, Overall and by Age Group.

Variable	Overall	13–24	25–34	35–44
**Age**				
13–24	435 (16.8)			
25–34	1,066 (41.1)			
35–44	1,094 (42.2)			
Gender***				
Male	2,005 (77.3)	373 (85.8)	830 (77.9)	802 (73.3)
Female	590 (22.7)	62 (14.3)	236 (22.1)	292 (26.7)
Race/ethnicity***				
White, non-Hispanic	650 (25.1)	61 (14.0)	273 (25.6)	316 (28.9)
Black, non-Hispanic	1,314 (50.6)	287 (66.0)	509 (47.8)	518 (47.4)
Hispanic	524 (20.2)	67 (15.4)	231 (21.7)	226 (20.7)
Other	107 (4.1)	20 (4.6)	53 (5.0)	34 (3.1)
HIV Transmission***				
IDU	161 (6.2)	7 (1.6)	50 (4.7)	104 (9.5)
HET	855 (33.0)	88 (20.2)	331 (31.0)	436 (39.9)
MSM	1,470 (56.7)	320 (73.6)	641 (60.1)	509 (46.5)
Other	109 (4.2)	20 (4.6)	44 (4.1)	45 (4.1)
Baseline CD4***				
<200	825 (31.8)	76 (17.5)	324 (30.4)	425 (38.9)
201–500	1,472 (56.7)	312 (71.7)	612 (57.4)	548 (50.1)
>500	298 (11.5)	47 (10.8)	130 (12.2)	121 (11.1)
**Baseline viral load (copies/ml)**	46,467	42,941	46,228	49,461
Median (IQR)	(12,400–135,000)	(12,400–115,178)	(11,610–136,104)	(13,000–150,277)
Initial cART Regimen***				
NNRTI	1,026 (39.5)	200 (46.0)	410 (38.5)	416 (38.0)
PI	863 (33.3)	92 (21.2)	337 (32.6)	424 (38.8)
Other	80 (3.1)	6 (1.4)	42 (3.9)	32 (2.9)
Unknown	626 (24.1)	137 (31.5)	267 (25.1)	222 (20.3)
Year of cART Initiation***				
2008	618 (23.8)	75 (17.2)	236 (22.1)	307 (28.1)
2009	596 (23.0)	90 (20.7)	248 (23.3)	258 (23.6)
2010	660 (25.4)	127 (29.2)	263 (24.7)	270 (24.7)
2011	721 (27.8)	143 (32.9)	319 (29.9)	259 (23.7)
**365-day gap between CD4 tests**				
0	1,857 (71.6)	318 (73.1)	756 (70.9)	783 (71.6)
1	610 (23.5)	102 (23.4)	261 (24.5)	247 (22.6)
2–4	128 (4.9)	15 (3.5)	49 (4.6)	64 (5.9)

Note: Entries are N and column percentage.

*** P-value for chi-squared test of association < 0.0001

Abbreviations: IDU = injection drug use; MSM = men who have sex with men; HET = heterosexual; cART = combination antiretroviral therapy

### Baseline CD4

For 2,173 patients (84%), the baseline CD4 occurred prior to the date of cART initiation; and for 422 (16%) the baseline CD4 occurred in the same week at cART initiation. Overall, the median number of weeks between baseline CD4 and ART initiation was 3 weeks prior (IQR 6 to 2 weeks prior). Baseline CD4 medians were 320 (IQR: 232–410) for ages 13–24, 293 (IQR: 158–403) for ages 25–34 and 258 (IQR: 106–384) cells/mm^3^ for ages 35–44, respectively. Proportions with baseline CD4 ≤ 200 were 0.17, 0.30, and 0.39 in respective age groups.

### Longitudinal CD4 trends

**Fig 1:**
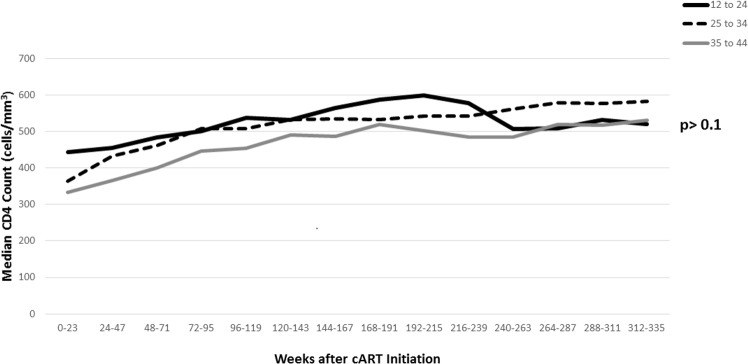
Unadjusted Median CD4 Levels, by Age Group and Weeks from cART Initiation.

Fig 1 shows observed (unadjusted) median CD4 levels by 24-week periods by age group (see S1 Table for estimates). Median CD4 levels in the first 24-week period post-cART initiation were highest among youth and lowest among older adults, consistent with differences in baseline CD4. In each age group, median CD4 tended to rise (with 4 exceptions) from one 24-week period to the next, up to 215 weeks from cART start. Youth attained a higher maximum than older groups. For youth, median CD4 levels declined from their maximum after week 215; decline in CD4 levels was less pronounced for adults.

Table 2 displays results of two mixed-model multivariate regression analyses of longitudinal changes in CD4. Random effects estimates appear in S2 Table. The set of CD4 outcomes for each patient began with the first CD4 test after cART initiation. Without adjusting for baseline CD4 (Model 1), CD4 levels (i.e., the linear slope) after cART start increased an average of 36.73 cells/mm^3^ per 24-week period for the 13–24 age group. The significant negative coefficient for squared time period (-2.10) indicates that the CD4 trajectory tended to diminish (i.e., become flatter) in later time periods for the youngest group. Interactions between age group and time (linear and quadratic) were not significant, with the exception of a significant positive quadratic term for the oldest age group, implying that the CD4 trajectory for the oldest group showed less of a downturn than that for younger persons.

**Table 2:** Mixed-model Regression Analysis of CD4 Levels.

Variable	coefficient (95% CI)	coefficient (95% CI)
	Model 1	Model 2
Time (24-week periods)	**36.73** (30.6, 42.9)	**38.98** (32.5, 45.5)
Time squared	**-2.10** (-2.6, -1.6)	**- 2.09** (-2.6, - 1.5)
**Age group**		
13–24	Reference	Reference
25–34	-23.47 (-49.2, 2.2)	-25.93 (-62.5, 10.6)
35–44	**-48.29** (-74.5, -22.1)	-27.15 (-63.2, 8.9)
Time*Age		
13–24	Reference	Reference
25–34	0.56 (-6.8, 7.9)	0.07 (-7.2, 7.4)
35–44	-4.37 (-11.7, 2.9)	-5.29 (-12.6, 2.0)
Time Squared*Age		
13–24	Reference	Reference
25–34	0.27 (-0.4, 0.9)	0.29 (-0.3, 0.9)
35–44	**0.76** (0.1, 1.4)	0.79 (0.2, 1.4)
**Baseline CD4 (cells/mm**^**3**^**)**		
≤200	Reference	Reference
201–500	——	221.46 (185.4, 237.5)
>500	——	**427.18** (374.9, 479.5)
Baseline CD4 * Age^**⊥**^		
201–500/ 25–34	——	32.66 (- 8.0, 73.3)
201–500/ 35–44	——	22.31 (-18.0, 62.6)
>500 / 25–34	——	**95.63** (36.1, 155.1)
>500 / 35–44	——	**120.43** (60.9, 180.0)
Baseline CD4*Time		
≤200	Reference	Reference
201–500	——	**-2.70** (-5.1, -0.2)
>500	——	**-7.62** (-11.7, -3.6)
**Year_gap**	**-24.12** (-33.5, -14.8)	**-25.69** (-35.0, -16.4)
**Viral Load Category (copies/mL)**		
< = 400	Reference	Reference
401–1000	-**36.51** (-44.1, -28.9)	**-34.71** (-42.2, -27.2)
>1000	**-93.28** (-98.3, -88.2)	**-93.94** (-98.9, -89.0)
**Gender**		
Male	Reference	Reference
Female	**39.39** (13.9, 64.91)	12.60 (-5.0, 30.2)
**Race/ethnicity**		
White	Reference	Reference
African-American	**-49.83** (-71.8, -27.8)	-14.22 (-29.5, 1.7)
Hispanic	**-49.06** (-74.0, -24.1)	- 9.45 (-26.7, 7.7)
Other	-24.78 (-67.9, 18.3)	-4.01 (-33.5, 25.5)
**HIV Transmission**		
IDU	Reference	Reference
HET	0.83 (-36.0, 37.6)	3.29 (-22.1, 28.7)
MSM	32.02 (- 4.3, 69.4)	16.46 (-8.6, 41.5)
Other	-26.51 (-79.4, 26.4)	4.15 (-32.5, 40.8)
**Year of cART Initiation**		
2008	Reference	Reference
2009	17.07 (-6.4, 40.6)	**17.40** (1.3, 33.5)
2010	**66.29** (42.7, 89.9)	**25.37** (9.1, 41.6)
2011	**136.35** (106.9, 165.8)	**44.36** (23.8, 64.9)
**Initial cART Regimen**		
NNRTI	Reference	Reference
PI	**-43.13** (-62.6, -23.7)	-10.5 (-23.9, 3.0)
Other	11.41 (-36.7, 59.5)	25.73 (-7.5, 59.0)
Unknown	**-71.87** (-99.7, -44.0)	-0.76 (-20.1, 18.6)
Constant	423.20	207.49

Note: Bolded coefficients are significant at p < .05. N = 25,276 CD4 measures, for 2,595 patients.

^⊥^Reference is the 13–24 age by CD4 ≤200 categories.

When baseline CD4 levels were also controlled (Model 2), the linear CD4 slope was 38.98 cells/mm^3^ per 24-week period for the 13–24 age group, with a quadratic component of -2.09. These values are close to those obtained in Model 1. Similarly, age group did not interact significantly with the linear time variable in Model 2. S3 Table shows mean CD4 levels predicted by Model 2 by age group and specific time point after cART initiation. Overall, CD4 trajectories over time were similar for youth and adults after adjusting for baseline CD4 level.

In addition, CD4 trajectories over time differed significantly as a function of baseline CD4. Compared to those with baseline CD4 ≤200, the linear slope was significantly lower by 2.70 units for patients with baseline CD4 201–500, and was 7.62 units lower for patients with baseline CD4>500. Thus, the overall rate of CD4 improvement declined as the baseline CD4 increased. S4 Table shows predicted CD4 levels by baseline CD4 category and specific time point.

Further, when patients had a gap in care, reflected in the time between successive CD4 tests of 365 days or longer, CD4 levels declined by 26 cells/mm^3^ in Model 2. In addition, compared to VL≤400, VL values between 401–1000 copies/mL were associated with a decrease of 35, and VL values above 1000 were associated with a decrease of 94 CD4 cells/mm^3^.

### Differences at time of cART initiation

In the regression models, coefficients for variables that are not interacted with time represent predicted differences immediately after cART initiation (i.e., time = 0). Without adjusting for baseline CD4 category (Model 1), patients aged 25–34 and 35–44 had significantly lower CD4 values than youth just after starting cART. In Model 2, due to including an interaction between age and baseline CD4, the main effect for age group pertains to age differences among those with baseline CD4 ≤200; in this group, age differences were not significant. The coefficients of the baseline CD4 by age interaction show that age differences were not significant among those with baseline CD4 201–500, but were significant among those with baseline CD4 >500. Each adult age group had significantly higher CD4 levels right after cART initiation than youth when baseline CD4 was > 500. To help visualize this pattern, S5 Table shows median CD4 levels by age group and baseline CD4 just after cART initiation predicted from Model 2 and also at 24 weeks.

In Model 1, there were significant gender, racial/ethnic, cART regimen, and HIV risk factor differences in CD4 levels at cART initiation. However, when baseline CD4 levels were controlled (Model 2) the magnitude of these differences diminished, with gender, race, and risk factor becoming nonsignificant. In both models, however, CD4 after cART initiation was higher among those who initiated cART after 2008.

### Trends in virologic suppression

Table 3 shows the proportion of patients in each age group who were virologically suppressed (≤400 copies/mL) in each 24-week period following cART initiation. The proportion increased between the first and second time periods for each age group, but, unlike CD4 levels, the proportion suppressed did not show a consistent trend subsequently and instead fluctuated within a few percentage points in each age group. The youngest age group began with a greater proportion suppressed than the older age groups, but by the ninth and tenth periods the older age groups had somewhat higher proportions with suppressed viral load.

**Table 3:** Proportion of Patients with Virologic Suppression (≤400 copies/mL), by Age Group and Weeks from cART Initiation.

	Age Group		
Weeks	13–24	25–34	35–44
0–23	0.69 (750)	0.64 (1,823)	0.61 (1,942)
24–47	0.78 (569)	0.78 (1,262)	0.76 (1,348)
48–71	0.76 (507)	0.78 (1,075)	0.76 (1,154)
72–95	0.74 (457)	0.80 (970)	0.78 (1,003)
96–119	0.80 (419)	0.79 (883)	0.81 (960)
120–143	0.78 (374)	0.76 (842)	0.82 (863)
144–167	0.77 (351)	0.77 (786)	0.82 (817)
168–191	0.76 (280)	0.78 (577)	0.80 (717)
192–215	0.75 (210)	0.81 (508)	0.79 (604)
216–239	0.73 (150)	0.79 (375)	0.81 (521)
240–263	0.75 (117)	0.77 (312)	0.78 (423)
264–287	0.71 (82)	0.81 (229)	0.83 (318)
288–311	0.88 (51)	0.72 (149)	0.83 (246)
312–335	0.70 (43)	0.77 (139)	0.82 (174)

Note: First cell entry is proportion with virologic suppression; second number is sample size. Observations in each cell represent number of VL tests during the time period. Observations in different rows are not mutually exclusive.

To examine age trends further, we used a mixed logistic regression model. (Table 4) The model was similar to that used for CD4, but baseline CD4 variables were removed. In each 24-week period, the odds of being virologically suppressed increased by 30%. The significant squared term indicates that the rate of increase diminished over time. Age groups did not differ significantly in the proportion suppressed at the initiation of cART, but the rate of increase was significantly greater for the oldest group, compared to youth.

**Table 4:** Mixed Longitudinal Regression of Viral Load Suppression.

Variable	Adjusted Odds Ratio (95% CI)
Time (24-week periods)	**1.30** (1.19, 1.42)
Time squared	**0.98** (0.97, 0.99)
**Age group**	
13–24	Reference
25–34	0.89 (0.64, 1.23)
35–44	**0.65** (0.47, 0.91)
Time*Age	
13–24	Reference
25–34	1.11 (0.99, 1.23)
35–44	**1.21** (1.09, 1.34)
Time Squared*Age	
13–24	Reference
25–34	0.99 (0.98, 1.01)
35–44	**0.99** (0.98, 0.99)
**Gender**	
Male	Reference
Female	**0.73** (0.56, 0.96)
**Race/ethnicity**	
White	Reference
African-American	**0.59** (0.46, 0.75)
Hispanic	0.84 (0.64, 1.11)
Other	1.13 (0.69, 1.83)
**HIV Transmission**	
IDU	Reference
HET	**1.59** (1.07, 2.36)
MSM	**1.91** (1.28, 2.82)
Other	0.89 (0.50, 1.58)
**Initial cART Regimen**	
NNRTI	Reference
PI	**0.55** (0.45, 0.68)
Other	0.84 (0.50, 1.43)
Unknown	1.04 (0.82, 1.31)

Note: Bolded coefficients are significant at p < .05. N = 25,380 VL measures, for 2,572 patients. 23 persons had no VL data after HAART initiation.

## Discussion

Analyses of a large cohort of young PLHIV receiving cART reveals three major findings: youth who start cART do so at higher CD4 counts compared to older adults; youth have better initial CD4 responses than older adults; and, possibly due to decreased adherence, youth’s CD4 response plateaus, allowing adults to reach similar CD4 levels after sufficient time has passed.

Specifically, the median CD4 levels of young PLHIV tended to rise consistently up to 215 weeks after cART initiation, however, the (adjusted) linear rate of increase did not differ significantly between youth (aged 13–24) and adults (aged 25–44). Those with higher baseline CD4 had higher subsequent CD4 at any point in time [37–39]. Higher viral load and gaps between CD4 tests of a year or longer were each associated with lower CD4 levels.

Studies that have compared the immune recovery of youth to that of adults in non-HIV settings have suggested that youth have a greater capacity to regenerate their immune system, likely as a result of their residual thymic capacity. [19,22,23] The relative CD4 recovery of nPHIV youth living with HIV is unclear; studies that have attempted to evaluate the question have tended to lump youth into older age groups or mixed nPHIV youth with perinatally HIV-infected youth, impairing assessment of the specific responses of nPHIV youth to cART. [21,24] [40] [41,42]

It is well established that the lower the CD4 at initiation of therapy, the lower CD4 threshold that one will be able to reach, as evidenced by the longitudinal data in this study. [38,43] Individuals with the lowest baseline CD4 values (≤200 cells/mm^3^), compared to those in the higher CD4 categories, had more rapid rises in CD4 counts (i.e., higher linear slope), but ultimately had lower CD4 levels at the end of follow-up. This finding likely relates to the reduced regenerative capacity in lower CD4 categories and the better preserved capacity to repopulate CD4 in higher CD4 categories, further underscoring the importance of early cART initiation. [12,44] Although youth may have an initial short-term advantage in reaching higher CD4 levels after cART initiation, the long-term difference (compared to older adults) is minimal, suggesting that treatment initiation for youth should not be delayed until CD4 counts decline. Additionally, the consequences of unchecked virologic replication, including the risk of secondary transmission and systemic sequelae of immune activation, likely outweigh any advantage of delaying therapy in this population. [45–47]

The virologic suppression rates in this study were higher than those previously reported in clinical studies of youth initiating cART. [7,8] Prior studies have shown lower virologic suppression rates for youth than adults. [8] These studies include a mix of observational and clinical trial data, and many examine older regimens (e.g., higher dose PI regimens) or less tolerable agents (e.g., efavirenz). [5,8] For the more contemporary regimens initiated during the study period between 2006 and 2012, we show that youth had similar virologic suppression rates as their adult counterparts. Given that regimens are becoming increasingly more streamlined, with combination tablets, tolerable agents, with higher barriers to resistance, this finding is promising and may improve confidence among providers to initiate cART earlier for youth.[13]

Ultimately, youth did not attain higher CD4 values than their adult counterparts. It is possible that the lack of continued improvement was related to increased non-compliance over time. In PACTG 381, Flynn et al reported decreased adherence, as measured by virologic suppression, from 59% to 49% to 24% over 24, 60, and 152 weeks on cART. [8,9] In contrast, the virologic suppression rates of youth remained similar to adults over the course of this study.

Some additional demographic characteristics were associated with higher CD4 after ART initiation in Model 1, specifically, female gender, white race, and initiating cART in the latter years of study (e.g., 2011 vs. 2008). However, these differences (except for year of cART initiation) were reduced by adjusting for baseline CD4, suggesting that baseline CD4 differences underlie some observed demographic associations.

Individuals with gaps in care had significantly lower CD4 levels compared to those who were consistently in care. In fact, each additional one year gap in care resulted in a 40 cells/mm^3^ reduction in CD4 levels, suggesting the importance of continued engagement in care and maintenance of cART, once initiated. This finding is consistent with other studies. [38,48,49] Short term gains while on therapy are not maintained when therapy is (probably) interrupted. [50,51] Additionally, the risk of virologic nonsuppression and the potential for rebound in immune activation and their potential sequelae provide additional evidence for measures supporting patient continuity of care. [45,46]

Our results should be interpreted with consideration of several important limitations. First, the number of youth under 18 was negligible, and our findings for youth may not generalize to those aged 13–17. Second, although our HIVRN demographics closely resemble those of PLHIV in the U.S. in general, our sample is not nationally representative. Third, mental health, substance abuse, socioeconomic status, and other psychosocial characteristics, which may be important factors that impact virologic suppression and by extension CD4 response, are not measured in the HIVRN and therefore could not be examined. Fourth, this analysis focuses on the response to cART initiation and does not examine regimen changes. Fifth, differences between CD4 outcomes of youth and those of adults ≥45 years old are likely pronounced, given the impact of ageing on the immune system. This was not the focus of this study, and we cannot comment on possible differences between youth and this older age group.

Finally, there was substantial attrition from the study over time, as is common in longitudinal investigations. Such attrition could potentially bias results if those who drop out are systematically different from those who remain (e.g., have lower or higher rates of CD4 improvement). The mixed-model analysis assumes that data are missing at random (and not the more restrictive missing completely at random assumption), and the regression model included several demographic factors that could be related to propensity to drop out, which may mitigate potential attrition biases.

In conclusion, youth followed in non-clinical-trials settings of HIVRN clinics achieved CD4 outcomes similar to adults. Despite youth’s residual thymic tissue and capacity of immune reconstitution, initiation of cART should not be delayed for youth with the expectation that residual capacity for immune reconstitution will result in greater CD4 responses that will be sustained. Initiating cART soon after diagnosis and maintaining virologic suppression are the most critical factors to enhancing CD4 outcomes, especially for youth who traditionally have higher nonadherence rates.[8,17,52–54]. Our data suggest that, even with their residual thymic capacity, youth and adults have similar immune recovery after initiating cART, adjusting for baseline CD4, and that emphasis should be placed on initiating cART as close to diagnosis as possible when CD4 counts are higher. Studies of mechanisms to support youth’s continued adherence to therapy and their impact on resultant virologic suppression are critical.

## Supporting information

S1 Table Unadjusted Median CD4 Levels, by Age Group and Weeks from cART Initiation. Cell entries are median CD4, number of observations, and number of unique patients. Number of observations exceeds number of patients in each time interval because one person can contribute multiple observations. Observations in different time periods are not independent; the same person can have data in multiple time periods.
(DOCX)

S2 Table Random Effects Parameters from Second Regression Model. Note: SD- standard deviation; corr- correlation.
(DOCX)

S3 Table Predicted (Adjusted) Mean CD4 Levels, by Age Group and 24-Week Periods from Baseline (Model 2). Note: Entries are mean CD4 levels predicted by regression model 2, averaging over other covariates.
(DOCX)

S4 Table Predicted (Adjusted) Mean CD4 Levels, by Baseline CD4 and 24-Week Periods from Baseline (Model 2). Note: Entries are mean CD4 levels predicted by regression model 2, averaging over other covariates.
(DOCX)

S5 Table Predicted mean CD4 levels by age group and baseline CD4 at Time of cART initiation and 24 Weeks after cART Initiation. (DOCX)
