# Supplementary material for: Comparing longitudinal CD4 responses to cART among non-perinatally HIV-infected youth versus adults: Results from the HIVRN Cohort
Source: PLoS One. 2017 Feb 9;12(2):e0171125. doi: 10.1371/journal.pone.0171125 (PMC5300758; doi:10.1371/journal.pone.0171125)
Supplement: S1 Table — Cell entries are median CD4, number of observations, and number of unique patients. Number of observations exceeds number of patients in each time interval because one person can contribute multiple observations. Observations in different time periods are not independent; the same person can have data in multiple time periods. (DOCX) [file pone.0171125.s001.docx]

**S1 Table. Unadjusted Median CD4 Levels, by Age Group and Weeks from cART Initiation**

|  | **Age Group** | | |
| --- | --- | --- | --- |
| **Weeks** | **13-24** | **25-34** | **35-44** |
| 0-23 | 443 (739) (435) | 363 (1,789) (1,066) | 334 (1,861) (1,094) |
|  |  |  |  |
| 24-47 | 454 (582) (343) | 432 (1,305) (796) | 363 (1,373) (821) |
|  |  |  |  |
| 48-71 | 486 (529) (323) | 465 (1,085) (704) | 398 (1,169) (745) |
|  |  |  |  |
| 72-95 | 500 (458) (282) | 509 (989) (655) | 443 (1,020) (658) |
|  |  |  |  |
| 96-119 | 538 (420) (273) | 506 (882) (620) | 451.5 (943) (630) |
|  |  |  |  |
| 120-143 | 537 (377) (260) | 526 (883) (587) | 487 (882) (599) |
|  |  |  |  |
| 144-167 | 565 (342) (235) | 528 (780) (555) | 485 (814) (567) |
|  |  |  |  |
| 168-191 | 587 (261) (186) | 525 (581) (451) | 510 (704) (512) |
|  |  |  |  |
| 192-215 | 597.5 (212) (146) | 542 (512) (377) | 501 (605) (428) |
|  |  |  |  |
| 216-239 | 576 (151) (115) | 539 (373) (281) | 476 (504) (356) |
|  |  |  |  |
| 240-263 | 498 (119) (83) | 560 (307) (229) | 481.5 (402) (274) |
|  |  |  |  |
| 264-287 | 509 (78) (60) | 586 (226) (178) | 517 (317) (225) |
|  |  |  |  |
| 288-311 | 539 (51) (36) | 583 (147) (114) | 519 (231) (163) |
|  |  |  |  |
| 312-335 | 520 (25) (20) | 582 (100) (83) | 527 (130) (99) |

Cell entries are median CD4, number of observations, and number of unique patients. Number of observations exceeds number of patients in each time interval because one person can contribute multiple observations.

Observations in different time periods are not independent; the same person can have data in multiple time periods.
