# Supplementary material for: Comparing longitudinal CD4 responses to cART among non-perinatally HIV-infected youth versus adults: Results from the HIVRN Cohort
Source: PLoS One. 2017 Feb 9;12(2):e0171125. doi: 10.1371/journal.pone.0171125 (PMC5300758; doi:10.1371/journal.pone.0171125)
Supplement: S2 Table — Note: SD- standard deviation; corr- correlation. (DOCX) [file pone.0171125.s002.docx]

**Supplementary Table 2. Random Effects Parameters from Second Regression Model**

| **Site Level** | **Estimate** | **95% CI** |
| --- | --- | --- |
| Intercept (SD) | 21.79 | 11.48 to 41.38 |
| **Person Level** |  |  |
| Intercept (SD) | 126.72 | 121.71 to 131.93 |
| Time (SD) | 44.02 | 41.70 to 46.47 |
| Time^2^ (SD) | 3.10 | 2.88 to 3.35 |
| Intercept/time (corr) | -0.12 | -0.19 to -0.05 |
| Intercept/time^2^ (corr) | 0.04 | -0.04 to 0.13 |
| Time/Time^2^ (corr) | -0.90 | -0.91 to -0.88 |

Note: SD- standard deviation; corr- correlation
