# Supplementary material for: Comparing longitudinal CD4 responses to cART among non-perinatally HIV-infected youth versus adults: Results from the HIVRN Cohort
Source: PLoS One. 2017 Feb 9;12(2):e0171125. doi: 10.1371/journal.pone.0171125 (PMC5300758; doi:10.1371/journal.pone.0171125)
Supplement: S3 Table — Note: Entries are mean CD4 levels predicted by regression model 2, averaging over other covariates. (DOCX) [file pone.0171125.s003.docx]

S3 Table. Predicted (Adjusted) Mean CD4 Levels, by Age Group and 24-Week Periods from Baseline (Model 2)

|  | **Age Group** | | |
| --- | --- | --- | --- |
| **24-Week Period** | **13-24** | **25-34** | **35-44** |
| 0 | 376 | 379 | 374 |
|  |  |  |  |
| 24 | 411 | 414 | 404 |
|  |  |  |  |
| 48 | 441 | 445 | 432 |
|  |  |  |  |
| 72 | 468 | 473 | 457 |
|  |  |  |  |
| 96 | 489 | 497 | 479 |
|  |  |  |  |
| 120 | 508 | 518 | 499 |
|  |  |  |  |
| 144 | 521 | 535 | 516 |
|  |  |  |  |
| 168 | 531 | 548 | 530 |
|  |  |  |  |
| 192 | 536 | 558 | 542 |
|  |  |  |  |
| 216 | 538 | 564 | 552 |
|  |  |  |  |
| 240 | 535 | 567 | 559 |
|  |  |  |  |
| 264 | 527 | 566 | 563 |
|  |  |  |  |
| 288 | 516 | 561 | 565 |
|  |  |  |  |
| 312 | 501 | 533 | 564 |

Note: Entries are mean CD4 levels predicted by regression model 2, averaging over other covariates.
