# Supplementary material for: Comparing longitudinal CD4 responses to cART among non-perinatally HIV-infected youth versus adults: Results from the HIVRN Cohort
Source: PLoS One. 2017 Feb 9;12(2):e0171125. doi: 10.1371/journal.pone.0171125 (PMC5300758; doi:10.1371/journal.pone.0171125)
Supplement: S4 Table — Note: Entries are mean CD4 levels predicted by regression model 2, averaging over other covariates. (DOCX) [file pone.0171125.s004.docx]

S4 Table. Predicted (Adjusted) Mean CD4 Levels, by Baseline CD4 and 24-Week Periods from Baseline (Model 2)

|  | **Baseline CD4 (cells/mm^3^)** | | |
| --- | --- | --- | --- |
| **24-Week Period** | **<200** | **201-500** | **500** |
| 0 | 186 | 430 | 703 |
|  |  |  |  |
| 24 | 221 | 462 | 730 |
|  |  |  |  |
| 48 | 253 | 491 | 754 |
|  |  |  |  |
| 72 | 281 | 517 | 775 |
|  |  |  |  |
| 96 | 306 | 540 | 793 |
|  |  |  |  |
| 120 | 329 | 559 | 808 |
|  |  |  |  |
| 144 | 347 | 575 | 819 |
|  |  |  |  |
| 168 | 363 | 588 | 827 |
|  |  |  |  |
| 192 | 375 | 598 | 831 |
|  |  |  |  |
| 216 | 384 | 604 | 833 |
|  |  |  |  |
| 240 | 390 | 607 | 831 |
|  |  |  |  |
| 264 | 393 | 607 | 826 |
|  |  |  |  |
| 288 | 392 | 604 | 818 |
|  |  |  |  |
| 312 | 388 | 597 | 806 |

Note: Entries are mean CD4 levels predicted by regression model 2, averaging over other covariates.
