# Supplementary material for: Comparing longitudinal CD4 responses to cART among non-perinatally HIV-infected youth versus adults: Results from the HIVRN Cohort
Source: PLoS One. 2017 Feb 9;12(2):e0171125. doi: 10.1371/journal.pone.0171125 (PMC5300758; doi:10.1371/journal.pone.0171125)
Supplement: S5 Table — (DOCX) [file pone.0171125.s005.docx]

**S5 Table. Predicted mean CD4 levels by age group and baseline CD4 at Time of cART initiation and 24 Weeks after cART Initiation**

| **Time=0** | | | |
| --- | --- | --- | --- |
|  | **Age Group** | | |
| **Baseline CD4** | **13-24** | **25-34** | **35-44** |
| <200 | 208 | 182 | 181 |
| 201-500 | 429 | 436 | 424 |
| >500 | 635 | 705 | 728 |

| **Time=1 (24 weeks)** | | | |
| --- | --- | --- | --- |
|  | **Age Group** | | |
| **Baseline CD4** | **13-24** | **25-34** | **35-44** |
| <200 | 245 | 219 | 213 |
| 201-500 | 463 | 470 | 454 |
| >500 | 664 | 734 | 753 |
